# Reduction in limb-movement complexity at term-equivalent age is associated with motor developmental delay in very-preterm or very-low-birth-weight infants

**DOI:** 10.1038/s41598-024-59125-0

**Published:** 2024-04-10

**Authors:** Myung Woo Park, Hyung-Ik Shin, Moon Suk Bang, Don-Kyu Kim, Seung Han Shin, Ee-Kyung Kim, Eun Sun Lee, Hyun Iee Shin, Woo Hyung Lee

**Affiliations:** 1 Department of Rehabilitation Medicine, Chung-Ang University Hospital, Chung-Ang University College of Medicine, Seoul, Republic of Korea; 2 Department of Rehabilitation Medicine, Seoul National University Children’s Hospital, Seoul National University College of Medicine, Seoul, Republic of Korea; 3 National Traffic Injury Rehabilitation Hospital, Yangpyeong, Republic of Korea; 4 Department of Pediatrics, Seoul National University Children’s Hospital, Seoul National University College of Medicine, Seoul, Republic of Korea; 5 Department of Pediatrics, Chung-Ang University Hospital, Seoul, Republic of Korea; 6 Biomedical Research Institute, Chung-Ang University Hospital, Seoul, Republic of Korea

**Keywords:** Complexity, General movements, Motor development, Pose estimation, Preterm, Sample entropy, Paediatric research, Predictive markers, Neurological disorders

## Abstract

Reduced complexity during the writhing period can be crucial in the spontaneous movements of high-risk infants for neurologic impairment. This study aimed to verify the association between quantified complexity of upper and lower-limb movements at term-equivalent age and motor development in very-preterm or very-low-birth-weight infants. Video images of spontaneous movements at term-equivalent age were collected from very-preterm or very-low-birth-weight infants. A pretrained pose-estimation model and sample entropy (SE) quantified the complexity of the upper- and lower-limb movements. Motor development was evaluated at 9 months of corrected age using Bayley Scales of Infant and Toddler Development, Third Edition. The SE measures were compared between infants with and without motor developmental delay (MDD). Among 90 infants, 11 exhibited MDD. SE measures at most of the upper and lower limbs were significantly reduced in infants with MDD compared to those without MDD (p < 0.05). Composite scores in the motor domain showed significant positive correlations with SE measures at most upper and lower limbs (p < 0.05). The results show that limb-movement complexity at term-equivalent age is reduced in infants with MDD at 9 months of corrected age. SE of limb movements can be a potentially useful kinematic parameter to detect high-risk infants for MDD.

## Introduction

The early detection of high-risk infants for neurologic impairment is crucial for timely intervention. The identification of risk factors in preterm or low birth weight infants during the neonatal period facilitates early detection, leading to the initiation of early intervention and consequently, improving neurodevelopmental outcomes^1,2^. The General Movement Assessment (GMA), which was developed by Prechtl et al. on infantile spontaneous movements, is recognized as a reliable and valid method for the early detection of high-risk infants^3–5^. It can evaluate writhing and fidgety movements in infants from birth to 5 months of corrected age with high sensitivity and specificity to predict developmental delay and cerebral palsy^2,6–8^. Despite its high-level evidence in clinical practice, its clinical availability can be limited due to a high dependence on certified assessors who are educated through training courses along with its subjective nature as a qualitative or semi-quantitative assessment^4,9,10^.

In the last decade, there have been rapidly growing trends in applying computer vision techniques to quantitatively analyze infantile spontaneous movements^11–15^. This was driven by the development of pose-estimation models that allow the identification and classification of human joints in images or videos^10^. The pose-estimation models can extract the positional coordinates of human joints from images or videos, which can serve as the basis for various kinematic analyses of infantile spontaneous movements in a quantitative manner. Moreover, in developing automatic assessment tools using machine learning algorithms to predict neurodevelopmental outcomes in high-risk infants, it is required to discover novel kinematic parameters based on the spatial–temporal characteristics of infantile spontaneous movements.

Complexity, which refers to spatial and temporal variability, is a hallmark that can be used to differentiate between normal and abnormal spontaneous movements in infants during the writhing period in GMA: gross movements of the neck, trunk, arms, and legs in high-risk infants usually manifest as monotonous, synchronized, or stiff^16,17^. Thus, complexity can be considered a major candidate for a key kinematic parameter in developing automated assessments for prognostication based on infantile spontaneous movements. The complexity of human movement data can be quantitatively computed using sample entropy (SE), which is a measure of the extent of signal regularity for time series data^18^. In a previous study, SE values of kinematic data from inertial sensors attached to lower limbs showed significant associations with a risk of developmental delay^19^. Our previous study also revealed significant associations between SE values of upper- and lower-limb movements estimated using a pose estimation model and early neurological development in preterm infants at 4 months of corrected age^15^.

Even though SE might be a promising novel kinematic parameter to indicate motor developmental delay (MDD), few studies have investigated whether the SE of infantile spontaneous movements is associated with developmental outcomes in high-risk infants for MDD. Therefore, the aim of this study was to verify the association between quantified complexity of the upper- and lower-limb movements at term-equivalent age and motor development in very preterm or very low birth weight infants.

## Methods

### Study design and population

In this prospective, longitudinal cohort study, infants who were born with a gestational age of < 32 weeks or with a birth weight < 1500 g were enrolled from two tertiary hospitals. Infants who were admitted to neonatal intensive care units between March 2019 and August 2022 were included in this study after informed consent was acquired from parents or legal guardians. Exclusion criteria were genetic disorders, major congenital malformations, or unstable medical conditions such as requiring cardiovascular support, active sepsis, or surgery that can affect infantile spontaneous movements. The infantile and maternal clinical characteristics are presented in Table 1. The results of GMA were determined by two certified assessors who were blinded to the clinical information of the infants. Infants with a birth weight below 1000 g, born before 29 weeks of gestational age, or with severe intraventricular hemorrhage on brain ultrasonography underwent brain magnetic resonance imaging at term-equivalent age. This study was approved by the institutional review boards of the two tertiary hospitals. It was performed in accordance with all relevant guidelines and regulations.

**Table 1:** Clinical characteristics.

	Normal (n = 79)	Motor developmental delay (n = 11)	p-value
**Infantile characteristics**			
Sex (female)	26 (32.9)	8 (72.7)	0.018*
Gestational age (weeks:days)	29:0 (2:6)	27:3 (2:3)	0.077
Postnatal age (weeks:days) at video recordings	11:0 (3:0)	12:3 (2:3)	0.151
Birth weight (g)	1149.2 (361.3)	1029.3 (383.0)	0.308
Global score of HINE	66.2 (7.7)	57.5 (10.1)	0.001*
Categories of GMA			< 0.001*
Poor repertoire	17 (21.5)	5 (45.5)	
Cramped synchronized	0 (0.0)	2 (18.2)	
Chaotic	1 (1.3)	2 (18.2)	
**Neonatal morbidities**			
1-Min Apgar score^†^	4.7 (2.0)	3.0 (1.9)	0.008*
5-Min Apgar score^†^	7.2 (1.8)	5.1 (1.8)	0.002*
Bronchopulmonary dysplasia (moderate to severe)	22 (27.8)	6 (54.5)	0.073
Antenatal steroid^†^	53 (67.9)	4 (36.4)	0.051
Periventricular leukomalacia	5 (6.3)	5 (45.5)	< 0.001*
Intraventricular hemorrhage (grade III–IV)	3 (3.8)	4 (36.4)	0.004*
Invasive ventilator use (days)	10.1 (15.9)	38.2 (31.2)	0.014*
Patent ductus arteriosus	42 (53.2)	10 (90.9)	0.022*
Treatments of patent ductus arteriosus			0.585
Pharmacological closure	11 (13.9)	3 (27.3)	
Surgical closure	11 (13.9)	1 (9.1)	
Retinopathy of prematurity (stage 3–5)	5 (6.3)	3 (27.3)	0.055
Sepsis	10 (12.7)	5 (45.5)	0.006*
Seizure	1 (1.3)	4 (36.4)	0.001*
**Maternal characteristics**			
Maternal preeclampsia	12 (15.2)	0 (0.0)	0.348
Gestational diabetes mellitus	10 (12.7)	0 (0.0)	0.604
Multiple gestations	46 (58.2)	7 (63.6)	1.000
Maternal chorioamnionitis	23 (29.1)	2 (18.2)	0.721

Values are presented as mean (standard deviation) or number of participants (percentage).

*HINE* Hammersmith Infant Neurological Examination, *GMA* General Movement Assessment.

*Statistically significant at p < 0.05.

### Motor developmental outcomes

The Bayley Scales of Infant and Toddler Development, Third Edition (BSID-III), was used to evaluate the motor development of infants at 9 months of corrected age by experienced occupational therapists who were blinded to the medical histories of the infants. The BSID-III is one of the most widely used developmental assessments to detect developmental delays in infants and toddlers^20^. Among five domains in BSID-III, including cognition, motor, language, socio-emotional, and adaptive behavior, only the motor domain was analyzed in this study. MDD was defined as a motor composite score < 80 on the BSID-III in conjunction with rehabilitation needs determined by clinicians.

### Video data acquisition and analyses

Smartphone RGB cameras were used to collect video images of the spontaneous movements of preterm infants at term-equivalent age. The camera was positioned with a view that focused on the entire body, including the most distal parts, such as fingers and toes. Therapists or parents performed video recordings of infants in the supine position without agitation, such as fussing or crying, for 3–5 min with at least 2 continuous min in accordance with GMA^21^. If the infant was discharged from the hospital before the term-equivalent age, the parents were asked to take a video at home using a smartphone camera and send it to the research team. From the video images of the infants, the positional coordinates of the twelve joints, including the shoulders, elbows, wrists, hips, knees, and ankles at both sides, were extracted with an open-source, convolutional neural network-based pose-estimation model, AlphaPose^22^. The sampling frequency of all video images was 24 frames per second. After removing the positional coordinates with confidence levels less than 0.5, which were regarded as measurement errors, these were interpolated with a locally weighted smoothing method. The values of joint angle and joint angular velocity were calculated from the positional coordinates of each joint. The joint angle refers to the angle that is formed by the positional coordinates of the corresponding joint and its two adjacent joints. The joint angular velocity was calculated using the symmetric difference quotient as the sequence of the finite differences of the joint angles. Detailed information for acquiring video images, obtaining and preprocessing positional coordinates, and calculating joint angles and joint angular velocity were described in our previous study^15^.

### Calculation of sample entropy

**Figure 1:**
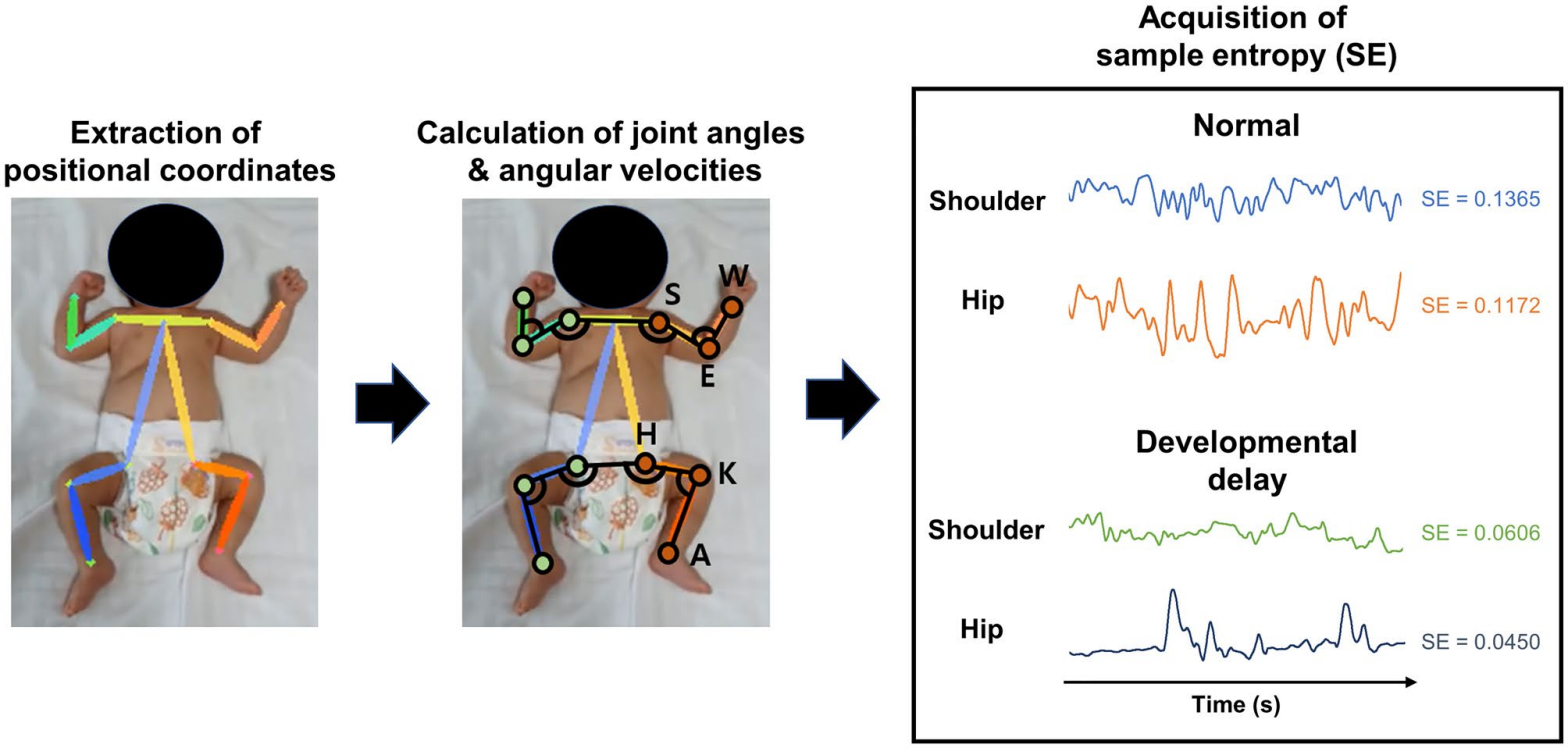
Overview of the acquisition process of sample entropy in very preterm or very low birth weight infants.

SE is an algorithm to determine the regularity of a time series of data based on the existence of patterns as a mathematical measure of the level of randomness^23^. A lower value of SE indicates more self-similarity and regularity in the time-series data. In this study, SE values were calculated from the joint angle and joint angular velocity at the upper and lower limbs to quantify the complexity of infantile spontaneous movements. SE was adopted as a complexity index for the spontaneous limb movements because it is a widely used algorithm to quantify the complexity of time-series data in human physiology and a more appropriate measure than approximate entropy in that it is largely independent of the time length of data^24,25^. Figure 1 illustrates the acquisition process of SE for the joint angle and joint angular velocity at the upper and lower limbs.

### Statistical analyses

Comparisons between infants with and without MDD were conducted using the Mann–Whitney *U*-test for continuous variables and the chi-squared test or Fisher’s exact test for categorical variables. Pearson correlation coefficients were obtained to analyze the correlations between the composite scores of BSID-III and SE values for joint angles and joint angular velocities. The SE values for joint angles and joint angular velocities were compared between preterm infants with and without MDD using the Student’s *t-*test or Mann–Whitney *U*-test. The significance level was set at *p* < 0.05. Statistical analyses were conducted using SPSS software (version 25; SPSS Inc, Chicago, IL, USA). SE values were calculated using the pracma package in R version 4.1.0 (The R Foundation, Vienna, Austria).

### Ethics approval and consent to participate

This study was approved by the institutional review boards of Seoul National University Hospital (1902-095-1011) and Chung-Ang University Hospital (2051-009-421). It was performed in accordance with all relevant guidelines and regulations.

## Results

### Clinical characteristics

Among a total of 90 consecutive infants, there were 11 infants with MDD in this study. Table 1 shows the infantile and maternal clinical characteristics. There were significant differences in the sex, global score of Hammersmith Infant Neurological Examination, categories of GMA, 1- and 5-min Apgar scores, periventricular leukomalacia, intraventricular hemorrhage, duration of the invasive ventilator use, presence of patent ductus arteriosus, history of sepsis, and history of seizure between infants with and without MDD (*p* < 0.05). The mean duration of video recordings of infantile spontaneous movements was 227.6 ± 103.4 s (infants with MDD, 279.9 ± 160.7 s; infants without MDD, 220.3 ± 91.9 s; *p* = 0.254).

### Sample entropy of infantile spontaneous movements

Table 2 shows the comparison results of the SE values for joint angle and joint angular velocity between infants with and without MDD. In all of the upper and lower limbs, the SE values of the joint angle were significantly reduced in infants with MDD compared to those without MDD. The SE values of the joint angular velocity in all of the upper and lower limbs except the right elbow were significantly lower in infants with MDD than those without MDD. The correlation coefficients between the motor composite scores of BSID-III and SE values of the joint angle and joint angular velocity are shown in Table 3. The SE values of the joint angle showed significant positive correlations with the composite scores of BSID-III at the right and left shoulder, right elbow, right hip, and right and left knee. The SE values of the joint angular velocity indicated significant positive correlations with the composite scores of BSID-III at the right shoulder, right hip, and right and left knee.

**Table 2:** Sample entropy measures for infantile spontaneous movements between infants with and without motor developmental delay.

		Normal (n = 79)	Motor developmental delay (n = 11)	p-value
Joint angle	Right shoulder	0.077 (0.036)	0.047 (0.021)	0.008*
	Left shoulder	0.076 (0.036)	0.043 (0.023)	0.004*
	Right elbow	0.066 (0.039)	0.036 (0.027)	0.014*
	Left elbow	0.071 (0.039)	0.040 (0.033)	0.015*
	Right hip	0.070 (0.034)	0.040 (0.022)	0.005*
	Left hip	0.069 (0.029)	0.041 (0.022)	0.004*
	Right knee	0.064 (0.031)	0.038 (0.025)	0.012*
	Left knee	0.066 (0.030)	0.037 (0.017)	0.002*
Joint angular velocity	Right shoulder	0.174 (0.069)	0.107 (0.047)	0.003*
	Left shoulder	0.170 (0.066)	0.109 (0.058)	0.005*
	Right elbow	0.151 (0.077)	0.093 (0.064)	0.020*
	Left elbow	0.159 (0.077)	0.091 (0.064)	0.006*
	Right hip	0.161 (0.062)	0.107 (0.041)	0.006*
	Left hip	0.162 (0.058)	0.111 (0.049)	0.006*
	Right knee	0.146 (0.059)	0.090 (0.036)	0.003*
	Left knee	0.149 (0.060)	0.086 (0.045)	0.001*

*Statistically significant at p < 0.05.

**Table 3:** Correlation coefficients between sample entropy measures for infantile spontaneous movements and motor composite scores of BSID-III.

		Correlation coefficients	p-value
Joint angle	Right shoulder	0.279	0.008*
	Left shoulder	0.271	0.010*
	Right elbow	0.260	0.013*
	Left elbow	0.190	0.073
	Right hip	0.237	0.024*
	Left hip	0.200	0.058
	Right knee	0.239	0.024*
	Left knee	0.254	0.016*
Joint angular velocity	Right shoulder	0.260	0.013*
	Left shoulder	0.206	0.051
	Right elbow	0.204	0.053
	Left elbow	0.168	0.113
	Right hip	0.239	0.023*
	Left hip	0.173	0.102
	Right knee	0.270	0.010*
	Left knee	0.230	0.029*

*BSID-III* Bayley Scales of Infant and Toddler Development, Third Edition.

*Statistically significant at p < 0.05.

## Discussion

The present study aimed to develop automated methods for measuring the complexity of infantile spontaneous movements, and to identify the relationship between the quantified complexity and motor development in high-risk infants. From the video images of spontaneous movements in very preterm or very low birth weight infants at term-equivalent age, the complexity of the upper and lower-limb movements was automatically quantified using deep learning-based pose estimation models and SE. The results revealed that SE values at most of the upper- and lower-limbs during spontaneous movements in infants with MDD were significantly lower than those without MDD. The SE values were also significantly correlated with the composite scores of the motor domain in the BSID-III in infants at 9 months of corrected age.

Our emphasis in this study was to identify novel endpoints that are calculated in an automated manner and exhibit significant associations with motor developmental outcomes rather than predicting the results of conventional evaluation methods such as GMA for newborns. Deep learning-based pose estimation algorithms were used to automatically transform video images of infantile spontaneous movements obtained using smartphone cameras into time-series data at multiple joints, including bilateral shoulders, elbows, hips, and knee joints. Complexity was determined as the main target to be analyzed because it is one of the most important characteristics associated with motor developmental delay or cerebral palsy and related to abnormal neurological outcomes in terms of the Hammersmith Infant Neurological Examination at 4 months of corrected age in our previous study^15^. To analyze the complexity of infantile spontaneous movements as time-series kinematic data, the SE was utilized as a time-independent measure considering the slightly variable time length of video images in the current study. Consequently, the current study expanded the results of our previous studies in that the obtained SE values at all joints showed significant or nearly significant associations with motor developmental outcomes at 9 months of corrected age, which may suggest that SE might be utilized as a feature variable in developing machine learning-based automatized models to predict developmental outcomes for determining early intervention in future studies.

The linear relationship between the complexity of early infantile movements in terms of SE and later motor development was evident in very preterm or very low birth weight infants in this study. The associations between movement complexity and motor development in high-risk infants are not surprising given that complexity is considered one of the most important characteristics in clinical evaluation methods such as GMA^16,17^. Infantile spontaneous movements are considered to provide a window for the early detection of developmental disorders: less variable and fluent movements with low spatial and temporal diversity during movements at the neck, trunk, arms, and legs may indicate a poor prognosis for development^16,26^. One of the main contributions of this study was to identify and demonstrate SE as a novel kinematic parameter to reflect the complexity of infantile spontaneous movements. Each limb movement was transformed into changes in the joint angle and joint angular velocity over time, which could be quantitatively measured by the degree of regularity using SE.

This study adopted writhing movements at term-equivalent age as the analysis target of importance because the complexity is strongly associated with MDD^2,4,27^. Infantile spontaneous movements may show spatiotemporal characteristics analogous to spontaneous activity in the neocortex of the developing brain (e.g. subplate) and possibly contribute to the acquisition of coordinated behavior through temporal sensorimotor learning without an explicit task or purpose^27,28^. According to the GMA, abnormal infantile spontaneous movements during the writhing period including poor repertoire, cramped synchronized, or chaotic movements at less than 2 months of corrected age may indicate the later occurrence of MDD or cerebral palsy^2,4,29^. Decreased complexity of infantile spontaneous movements is one of the major findings in these abnormal writhing movements and might be associated with impaired neural activity in the developing brain^4,27^. Whereas abnormalities in fidgety movements at 3–5 months of corrected age have higher predictive values than those in writhing movements, which were usually observed until 2 months of corrected age in previous studies^2,4^, it is clinically beneficial to analyze writhing movements when establishing management and therapeutic strategies at an earlier age. Additionally, tracking and analyzing writhing movements can have a technical advantage in that these are usually exhibited as a larger amplitude than fidgety movements, which are usually shown with a small amplitude and variable acceleration of small limb movements that might be too small to be analyzed.

Several previous studies have quantitatively analyzed infantile movements using different methodologies: inertial sensors with linear and/or nonlinear analyses^30–32^, kinematic analyses of lower-limb movements in video images (e.g. frequency, amplitude, phase duration)^33–35^, and SE of infantile movements based on inertial sensors^19^ or the center of pressure using force plate^32,33^. Particularly, SE is a commonly used method along with approximate entropy to quantify the complexity of physiological data including human movements by mathematically measuring the regularity of the degree of time-series data^25^. The results of this study showed that SE values of the bilateral upper- and lower-limb movements were lower in infants with MDD than in those without MDD. Because lower values of SE indicate more regular or repetitive patterns in time-series data, the results can be interpreted as more monotonous spontaneous movements of the bilateral upper and lower limbs in infants with MDD, possibly attributable to injuries of neural substrates^19^. These findings are generally consistent with those of previous studies which showed a low complexity of infantile movements, in terms of sample or approximate entropy, was closely related to poor developmental outcomes^19,36^. The low complexity of spontaneous lower-limb movements measured by multiple wearable sensors and postural sway in early sitting evaluated with a force plate was observed in infants with developmental delay in these previous studies.

There were several limitations in this study. First, the sample size of infants with MDD was relatively small compared to that of infants without MDD. Normative or cut-off values of SE values in high-risk infants were not investigated due to the small sample size, especially for infants with MDD. Larger cohort studies of neonatal populations are warranted to demonstrate the normative or cut-off values to differentiate high-risk infants who are candidates for early intervention. Second, the motor developmental outcomes of infants were assessed at 9 months of corrected age, which may be relatively young to identify their actual motor skill competence. However, because motor developmental delay was defined as low scores on the BSID-III along with clinical judgments on the need for rehabilitation in this study, it might be sufficiently indicative of determining early intervention. It is necessary to investigate whether the complexity of infantile spontaneous movements is associated with long-term developmental outcomes in future studies. Third, data acquisition and kinematic analyses were conducted solely on two-dimensional images of infantile spontaneous movements in very preterm or very low birth weight infants in this research. Even though this approach enabled to facilitate easier data acquisition using readily available devices such as conventional or smartphone RGB cameras, it could not incorporate three-dimensional analyses to analyze a broad range of movements, including antigravity movements which were previously reported to be associated with motor developmental outcomes in previous studies. For further studies aiming to develop automated, accurate, and robust methods for evaluating infantile spontaneous movements, the integration of three-dimensional limb movement data can be helpful to capture the comprehensive spectrum of movements and enhance validity regarding motor developmental outcomes.

In conclusion, this study demonstrated that the complexity of infantile spontaneous movements can be automatically quantified using a deep learning-based pose estimation model and SE. Moreover, the complexity of limb movements in very preterm or very low birth weight infants at the term-equivalent age was reduced in infants with MDD at 9 months of corrected age. The SE of the limb movements might be a useful kinematic parameter to detect high-risk infants for MDD. The complexity in terms of SE might have the potential to complement traditional evaluation methods such as GMA, by providing additional information on abnormalities of infantile spontaneous movements. Future research is warranted, focusing on larger cohorts with long-term follow-up of high-risk infants to establish normative values and to develop automatized models to predict developmental outcomes in clinical practice.

## Data Availability

The datasets used and/or analyzed during the current study are available from the corresponding author on reasonable request.
